# Trends and drivers of government health spending in sub-Saharan Africa, 1995–2015

**DOI:** 10.1136/bmjgh-2018-001159

**Published:** 2019-01-13

**Authors:** Angela E Micah, Catherine S Chen, Bianca S Zlavog, Golsum Hashimi, Abigail Chapin, Joseph L Dieleman

**Affiliations:** Institute for Health Metrics and Evaluation, University of Washington, Seattle, Washington, USA

**Keywords:** government health spending, sub-Saharan Africa, domestic health spending, government health expenditure

## Abstract

**Introduction:**

Government health spending is a primary source of funding in the health sector across the world. However, in sub-Saharan Africa, only about a third of all health spending is sourced from the government. The objectives of this study are to describe the growth in government health spending, examine its determinants and explain the variation in government health spending across sub-Saharan African countries.

**Methods:**

We used panel data on domestic government health spending in 46 countries in sub-Saharan Africa from 1995 to 2015 from the Institute for Health Metrics and Evaluation. A regression model was used to examine the factors associated with government health spending, and Shapley decomposition was used to attribute the contributions of factors to the explained variance in government health spending.

**Results:**

While the growth rate in government health spending in sub-Saharan Africa has been positive overall, there are variations across subgroups. Between 1995 and 2015, government health spending in West Africa grew by 6.7% (95% uncertainty intervals [UI]: 6.2% to 7.0%) each year, whereas in Southern Africa it grew by only 4.5% (UI: 4.5% to 4.5%) each year. Furthermore, per-person government health spending ranged from $651 (Namibia) in 2017 purchasing power parity dollars to $4 (Central African Republic) in 2015. Good governance, national income and the share of it that is government spending were positively associated with government health spending. The results from the decomposition, however, showed that individual country characteristics made up the highest percentage of the explained variation in government health spending across sub-Saharan African countries.

**Conclusion:**

These findings highlight that a country’s policy choices are important for how much the health sector receives. As the attention of the global health community focuses on ways to stimulate domestic government health spending, an understanding that individual country sociopolitical context is an important driver for success will be key.

**Key questions**

**What is already known?**

- National income and government fiscal space are important determinants of how much a country’s government spends on health.

**What are the new findings?**

- Country choices irrespective of income level, government fiscal space or population structure explain more of the level of government health spending observed in a country.
- Individual country characteristics such as good governance or the perceived levels of corruption in the public sector are important factors for understanding differences in government health spending across sub-Saharan African countries.

**What do the new findings imply?**

- For countries in sub-Saharan Africa, as the attention of the global health community focuses on ways to stimulate domestic government health spending, an understanding of individual country sociopolitical context will be most critical.
- Interventions that find a way to promote country ownership of the health-related Sustainable Development Goals may yield greater success in generating more spending on health from the government in support of the goals.

## Introduction

Government health spending is the primary source of health funding throughout the world. In 2015, government health spending made up 59.7% (59.2%–60.0%) of the total health spending globally, although it was only 34.4% (33.5%–35.2%) in sub-Saharan Africa.^1^ While the devastating effects of the HIV/AIDS epidemic and other diseases drew a great deal of attention and external funding to the subregion starting from the mid-1990s, recent changes have led to a plateauing of external resources for health.^2^ Additionally, estimates of the cost of attaining the health-related Sustainable Development Goals (SDGs) have led to a renewed sense of urgency to rally and leverage all possible resource avenues.^3^ These circumstances have revitalised an interest in understanding and tracking domestic government health spending in low-income and middle-income countries, and in sub-Saharan Africa in particular, as government health spending, both per capita and as a per cent of the total health spending in the region, remain among the lowest globally.

While there is an extensive literature tracking the growth and determinants of health spending in high-income countries, especially countries belonging to the Organisation for Economic Co-operation and Development, the literature focusing on the determinants of health spending in low-income and middle-income countries is relatively limited.^4–11^ There are databases from international agencies and research institutes that track and characterise cross-country estimates of health spending globally and government health spending in particular.^12–16^ Also, there exist in the literature a few studies on the determinants of government health spending in low-income and middle-income^17–21^ countries. The studies on government spending on health have examined factors such as national income, government fiscal space, population structure, health burden or disease prevalence, and health system characteristics. Findings from these studies show that national income and government fiscal space are important determinants of government health spending.^8–11 20 22 23^ In several studies, no statistically significant association is found between government health spending and population structure.^20 21 24^ The findings on the association between government health spending and health system characteristics have been mixed.^20 25^ The majority of existing studies have focused on quantifying the magnitude of the change in government health spending associated with a change in each factor, but this evidence is not indicative of how much of the cross-country and time variation in government health spending is explained by each factor. This gap is important to fill because while the estimated association between a factor and government health spending may be large, the size of the change in the factor over time may be small, such that this factor may actually contribute very little to explaining the variation in government health spending. A decomposition analysis of associated factors provides evidence on the contributions of each factor to explaining the observed variation. To this end, there are no studies the authors are aware of that have decomposed the explanatory power of the various factors associated with government health spending in these settings.

This study aims to contribute to filling the gap in knowledge of government health spending in sub-Saharan Africa by characterising the nature of government health spending in sub-Saharan African countries, examining the factors associated with government health spending in these settings, and most importantly highlighting the share of the explained variation in government health spending by these factors. The study focuses on sub-Saharan African countries in particular because, although we observe very high burden of diseases such as HIV and malaria, many countries in this subregion are among the countries with the lowest levels of government health spending globally. Therefore, such an analysis is timely and important because it will provide information on the main drivers of government health spending that policymakers may use to guide the development of strategies to meet country-specific health goals and the global SDGs.

## Methods

### Data

We used health spending data extracted from the Institute for Health Metrics and Evaluation’s Global Health Spending database.^12^ These health spending estimates are based on health spending by source data obtained from the WHO Global Health Expenditure Database, national health accounts and project-level data on development assistance for health.^13 26^ These data disaggregate total health spending into four mutually exclusive categories—domestic government health spending, prepaid private spending, out-of-pocket spending and development assistance for health—for 188 countries from 1995 through 2015. In this study, we limited the data set to 46 countries in sub-Saharan Africa, which are listed in table 2. We define domestic government spending on health as the aggregate value of transfers from government domestic revenue allocated to health purposes, social insurance contributions and compulsory prepayment.

We extracted gross domestic product (GDP) and general (all-sector) government spending (GGE) estimates from the same database. These data are based on data obtained from the International Monetary Fund, the World Bank, the United Nations, the Maddison Project and Penn World Tables database. Data from the various sources were combined using regression methods previously developed for producing a complete GDP time series.^27^ The GDP, GGE and health spending data are all reported in inflation-adjusted 2017 purchasing power parity adjusted dollars. We extracted data on tax revenue mobilisation from the Organisation of Economic Cooperation Global Revenue database and data on perceptions of corruption in the public sector from Transparency International.^28 29^ Additionally, we extracted demographic data for the 46 African countries from the Global Burden of Diseases, Injuries, and Risk Factors Study (GBD) 2016 database.^30^

We also used subregional classification from the GBD 2016 study^30^ to categorise the data in order to generate subregional rate of growth estimates. We calculated these subregional rates by aggregating national spending such that the rate is reflective of the subregion as a whole, rather than the average of its constituents.

### Examining the factors associated with government health spending

To examine the determinants of government health spending, we considered the following factors: national income, general government spending, development assistance for health, government tax revenue, perception of corruption in the public sector, time trend and population structure. With the exception of the measure for sociopolitical context—perception of corruption in the public sector—that is different from what is used in the existing literature, all the other factors have been included in previous studies on the determinants of health spending.^8–11 19 20 31 32^

The hypothesised associations between the included covariates and government health spending are based on evidence from previous literature. We hypothesised a positive association of government health spending with national income.^8 32^ In settings with more resources, more resources can be put towards the health sector to foster a healthy population that can support economic growth. Where the coefficient on income is positive but less than 1, it aligns with current view that health is a necessity good.^5^ Similarly, we hypothesised a positive association between the share of national income that is government spending and government health spending.^32^ As the share of the national income that is general government spending increases, more resources can trickle down to the health sector. We also hypothesised that the relationship between the flow of external resources for health and government health spending is negative.^31^ This implies that as more external resources flow into the health sector, less money flows from the domestic government to the health sector. We hypothesised a positive relationship between the population structure and government health spending, as well as between time and government health spending.^20^ Lastly, we hypothesised a positive relationship between the perception of corruption in the public sector and government health spending.^31^ Countries with a high perception of corruption are countries within which corruption levels are low and so fewer resources are misappropriated, leaving more resources in public coffers to be allocated.

National income was measured as per-person gross domestic product (GDPpc); the dependency ratio (Nonworkpop:Workpop), which represents the ratio of the non-working population (adults over 65 years and children under 15 years) to the working population (individuals 15–65 years), captured the population structure; development assistance for health received per person (DAHpc), general government spending as a share of the gross domestic product (GGE/GDP) and tax revenue as a share of the gross domestic product (TAXREV/GDP) were included to measure external assistance and government fiscal space, respectively. For the tax revenue as a share of GDP, the average proportion was used for country years with missing observations. Perception of corruption in the public sector (PCOR) was measured as an index on a scale from 0 to 100, where 0 equals the highest level of perceived corruption and 100 equals the lowest level of perceived corruption. The last available data point for the corruption perception index was held constant to fill subsequent years where there was missing country years of data. All variables were log-transformed. Country-level fixed effects were included to control for unobserved, time-invariant, country-specific factors. Summary statistics of all measures included in the analysis are shown in table 1.

**Table 1:** Descriptive statistics, 1995–2015

Variable	Mean	SD	Min	Max	Countries (n)	Observations (n)
Government health spending per capita	74.06	122.28	1.00	651.00	46	966
Gross domestic product per capita	3662.08	5450.12	274.83	45 933.21	46	966
Development assistance for health per capita	29.15	39.00	0.00	446.00	46	966
General government spending per gross domestic product	0.21	0.08	0.04	0.77	46	966
Tax revenue per gross domestic product	14.85	7.24	0.57	58.41	36	756
Dependency ratio	0.43	0.04	0.27	0.50	46	966
Corruption perception index	28.55	9.91	7.00	65.00	46	966

$$\begin{array}{ll} GHESpc_{it} & =\beta_{1}\left(GDPpc_{it}\right)+\beta_{2}{\left(\frac{GGE}{GDP}\right)}_{it}\\ & +\;\beta_{3}\left(DAHpc_{it}\right)+\beta_{4}{\left(\frac{Nonworkpop}{Workpop}\right)}_{it}+\beta_{5}{\left(\frac{Taxrev}{GDP}\right)}_{it}\\ & +\beta_{6}\left(PCOR_{it}\right)+\gamma_{t}+\nu_{i}+\varepsilon_{it} \end{array}$$

where *GHESpc*_*it*_ is government health spending per person in country *i* at time *t*. *β*_*1*_ *–* *β*_*6*_ are the coefficients on the relevant covariates listed previously, *γ*_t_ is the year trend, *ν*_*i*_ is the country fixed effects that capture the characteristics particular to the *i*th country which are time-invariant, and ε_it_ is the error term. Robust SEs clustered at the country level were applied.

Furthermore, a first differences model was also estimated. First differences models are unbiased and consistent if country fixed effects are correlated with the other independent variables. When there is serial correlation in the error term, the first difference is a more efficient estimator.^33^

### Decomposing the explained variation in government health spending

The results of the regression analysis report elasticities that quantify how the changes in each factor are associated with changes in government health spending. Those results do not quantify how much each factor explains the variation across country and time in government spending. While an estimated association could be relatively large, the actual change in the factor may have been relatively small in magnitude and that factor may not have explained much of the variation in observed government health spending. To quantify how much variation in government health spending each factor explains, we completed a Shapley decomposition analysis. This analysis splits the explained variance (R^2^) of the government health spending into contributions from each of the factors considered in this analysis. All analyses were completed in Stata V.13.^34^

## Results

**Figure 1:**
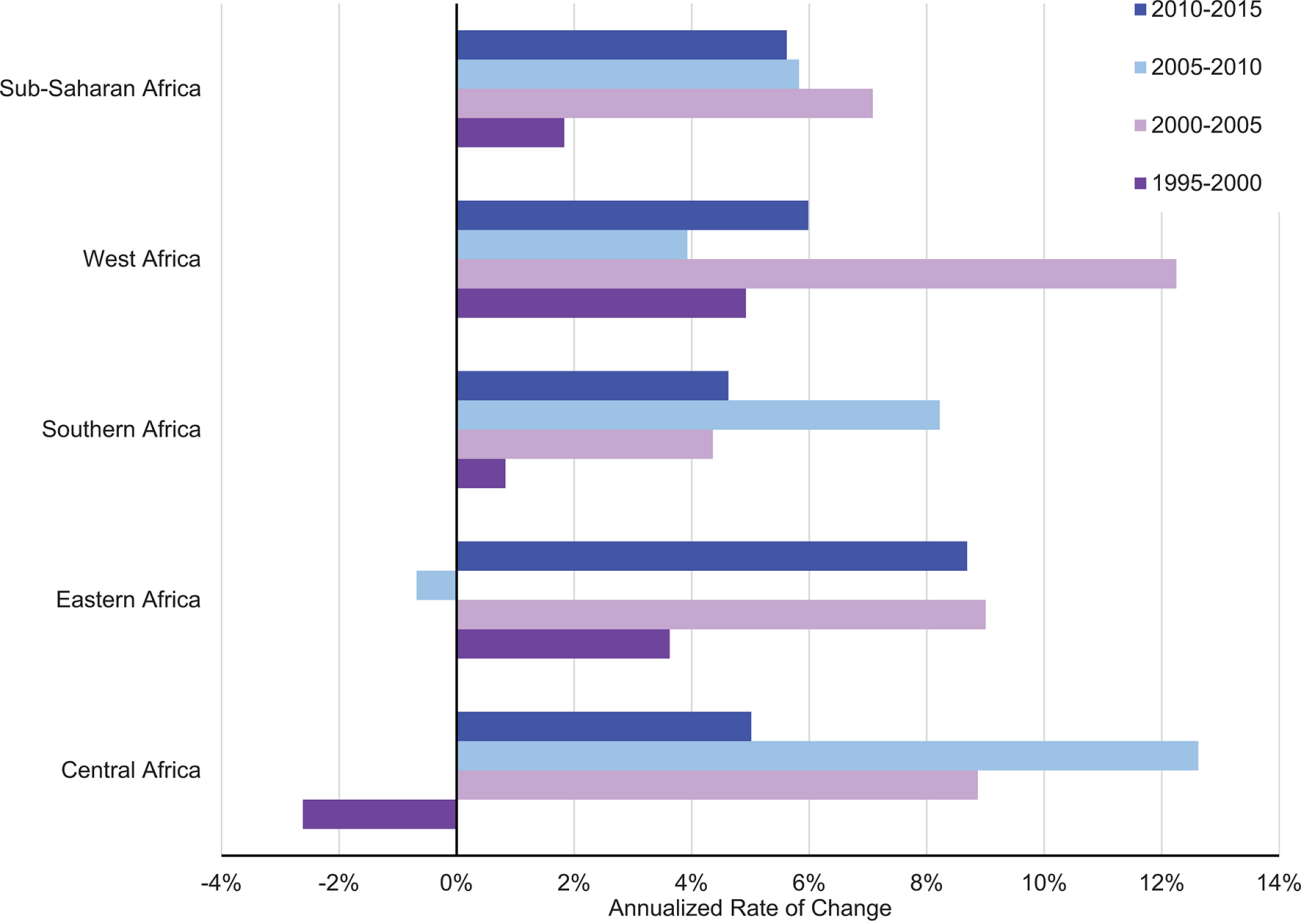
Annualised growth in total government health spending by sub-Saharan African subregions.

Figure 1 presents the annualised rate of growth in total government health spending across all countries in sub-Saharan Africa in 5-year intervals beginning 1995. Since 2000, government health spending has grown by more than 4% each year, with the highest period of growth being from 2000 through 2005. While the overall growth rate has shown positive year-on-year increase over the entire period, the highest periods of growth across all the subgroupings—East, West, Central and South—were observed between 2000 and 2015. West and Central Africa report growth rates as high as 12% during the periods 2000–2005 and 2005–2010, respectively. Interestingly, year-on-year growth in government health spending decreased in Central Africa from 1995 through 2000 and in Eastern Africa from 2005 to 2010.

Table 2 shows the total government health spending in each country, government health spending relative to population size, general government spending, national income and total health spending in 2015. For each country, these five metrics of government health spending highlight a different aspect of the value of government contribution to the health sector. While in some countries, like Namibia and Botswana, the total amount of government health spending is relatively low compared with other countries when put in the context of their relative population size, general government spending, total health spending and the size of their economy, they spend relatively more compared with other sub-Saharan African countries. There are also countries like South Africa that have relatively higher government health spending on all the metrics compared, while countries like Guinea, Eritrea and Benin fare relatively lower on all metrics compared. In terms of their relative population size, Namibia, South Africa and Botswana have the highest government health spending per person. Central African Republic, Somalia and Eritrea spend the smallest amount per person. Relative to each country’s overall general government spending, Namibia, South Africa and eSwatini have the highest share going to health. The Democratic Republic of Congo, South Sudan and Eritrea have the lowest share of government spending on health relative to the size of their overall government spending. Related to the share of national income that is spent as government health spending, Namibia, eSwatini and Lesotho have the highest share, while Nigeria, Central African Republic and Equatorial Guinea have the lowest shares relatively. As a percentage of total health spending, in Namibia, eSwatini and Cape Verde, at least 60% is financed from government source.

**Table 2:** Metrics for government health spending for sub-Saharan African countries, 2015

Country	Government health spending (thousands of 2017 PPP)	Government health spending per capita (2017 PPP)	Government health spending per general government spending (%)	Government health spending per gross domestic product (%)	Government health spending per total health spending (%)
Angola	$2 933 803.5	$117.0	5.8	1.5	59.4
Benin	$187 436.2	$17.0	3.6	0.8	20.7
Botswana	$1 274 363.5	$564.0	11.4	3.3	55.3
Burkina Faso	$506 926.2	$28.0	7.1	1.6	29.8
Burundi	$235 001.9	$21.0	9.9	2.5	31.3
Cameroon	$538 233.8	$23.0	4.4	0.7	14.7
Cape Verde	$119 298.3	$220.0	13.4	3.3	61.8
Central African Republic	$19 668.8	$4.0	5.3	0.6	14.3
Chad	$404 422.8	$29.0	9.1	1.2	28.2
Comoros	$12 969.3	$17.0	4.2	1.1	13.0
Congo	$400 116.3	$87.0	3.9	1.4	48.1
Congo, Democratic Republic of	$538 457.6	$7.0	2.1	0.7	15.9
Côte d'Ivoire	$1 012 413.1	$45.0	6.5	1.2	34.4
Djibouti	$81 419.6	$85.0	5.6	2.4	57.8
Equatorial Guinea	$194 345.8	$239.0	2.1	0.6	21.9
Eritrea	$52 131.8	$10.0	2.4	0.8	24.4
eSwatini	$564 245.2	$427.0	15.0	4.6	61.6
Ethiopia	$1 694 126.4	$17.0	7.1	1.0	21.0
Gabon	$495 711.6	$287.0	7.0	1.6	58.9
Gambia	$97 206.2	$49.0	12.6	2.8	34.8
Ghana	$2 572 307.8	$93.0	8.7	2.2	38.4
Guinea	$150 576.4	$12.0	3.6	0.8	11.8
Guinea-Bissau	$55 778.6	$30.0	13.5	1.9	24.8
Kenya	$2 589 042.5	$57.0	8.7	1.8	30.5
Lesotho	$295 930.8	$140.0	9.6	4.4	53.4
Liberia	$49 533.4	$11.0	5.3	1.2	2.3
Madagascar	$801 074.1	$33.0	7.8	2.2	42.3
Malawi	$451 964.2	$26.0	9.5	2.3	19.3
Mali	$295 927.3	$17.0	3.7	0.9	15.5
Mauritania	$286 183.1	$72.0	6.5	1.8	39.1
Mozambique	$308 546.8	$11.0	2.8	0.9	15.3
Namibia	$1 592 759.3	$651.0	17.8	5.6	63.0
Niger	$328 105.4	$17.0	5.5	1.7	25.4
Nigeria	$6 308 599.5	$35.0	6.4	0.6	16.2
Rwanda	$424 036.3	$36.0	9.3	1.9	24.2
Sao Tome and Principe	$19 937.6	$103.0	11.7	3.1	47.7
Senegal	$480 455.1	$32.0	5.6	1.3	26.9
Sierra Leone	$148 690.0	$23.0	9.3	1.6	9.3
Somalia	$50 506.7	$5.0	6.6	0.9	11.9
South Africa	$31 316 138.0	$594.0	17.4	4.4	53.6
South Sudan	$288 707.6	$22.0	2.2	0.7	27.2
Tanzania, United Republic of	$3 122 955.5	$59.0	12.1	2.1	36.6
Togo	$194 832.4	$27.0	7.9	1.8	28.1
Uganda	$860 166.2	$22.0	8.1	1.0	13.8
Zambia	$1 226 128.4	$76.0	9.6	1.9	31.5
Zimbabwe	$748 157.0	$48.0	8.3	2.2	25.1

PPP, purchasing power parity.

Table 3 highlights the results from the country fixed-effects and first difference government health spending regressions. Columns 1 through 5 show the results of the fixed-effects regression with relevant variables added successively. Columns 6 and 7 report the results with corrected Driscoll and Kraay SEs due to cross-dependence. Columns 8 and 9 show the results from the first difference models. Results from specification tests, autocorrelation and unit roots are reported in online supplementary appendix 1. A 10% increase in the perception of corruption in the public sector is associated with a 2.3% increase in government health spending per person. This result is robust to the inclusion of time fixed effects (columns 3 and 4), additional measures of fiscal space (columns 5 and 7), and remains positive but insignificant in the first difference model. GDP shows a positive and significant association with government health spending. The income elasticity is observed to be below 1 (0.66, 95% CI 0.49 to 0.83). A 10% rise in government spending as a share of national income is associated with a 5.3% (0.53, 95% CI 0.31 to 0.75) increase in government health spending. The flow of external resources for health and government health spending are not statistically significantly associated. The dependency ratio, which captures the burden on the working population for the vulnerable—young and older population—is also non-significantly associated with government health spending. The intracluster correlation shows that over 87% of the differences in government health spending are based on differences across countries.

10.1136/bmjgh-2018-001159.supp1 Supplementary data

**Table 3:** Regressions for government health spending per capita

	(1)	(2)	(3)	(4)	(5)	(6)	(7)	8 (FD)	9 (FD)
GDP per person	0.659*** (0.092)	0.609*** (0.081)	0.657*** (0.079)	0.659*** (0.084)	0.638*** (0.089)	0.659*** (0.068)	0.638*** (0.062)	0.624*** (0.071)	0.634*** (0.079)
Development assistance for health	−0.003 (0.020)	0.000 (0.019)	0.014 (0.020)	0.011 (0.022)	0.005 (0.030)	0.011 (0.012)	0.005 (0.011)	−0.004 (0.007)	−0.010 (0.008)
Government spending per GDP	0.528*** (0.103)	0.492*** (0.110)	0.513*** (0.107)	0.530*** (0.111)	0.478*** (0.132)	0.530*** (0.063)	0.478*** (0.072)	0.141*** (0.029)	0.157*** (0.035)
Dependent population	−0.427 (0.681)	−0.425 (0.664)	−0.784 (0.747)	−0.772 (0.758)	−0.765 (0.876)	−0.772* (0.378)	−0.765* (0.382)	−0.510 (0.612)	−0.811 (0.658)
Corruption perception index		0.225** (0.096)	0.226** (0.095)	0.241** (0.113)	0.342** (0.150)	0.241** (0.089)	0.342*** (0.085)	−0.005 (0.039)	0.017 (0.043)
Tax revenue per GDP					0.207* (0.115)		0.207*** (0.068)		0.009 (0.027)
Year			−0.006 (0.005)	−0.004 (0.004)	−0.006 (0.005)	−0.001*** (0.000)	−0.001*** (0.000)		
Constant	−1.069* (0.636)	−1.494** (0.637)	9.777 (8.789)	5.255 (7.455)	8.364 (9.260)			−0.013 (0.015)	−0.019 (0.018)
R^2^ within	44.29	45.55	46.13	47.29	51.42			15.60	17.50
F-statistic	28.77	28.40	28.99	23.15	42.14	22 914.14	6733.37	15.43	20.24
Year FEs				Yes	Yes	Yes	Yes	Yes	Yes
Regular dummies								Yes	Yes
CD	No	No	No	Yes	Yes	No	No	No	No
n (groups)	46	46	46	46	36	46	36	46	36
n (observation)	954	954	954	954	749	954	749	903	710

*** p<0.001, **p<0.05, *p<0.1

CD, Cross dependence; FD, First difference; FE, Fixed effects; GDP, gross domestic product.

**Figure 2:**
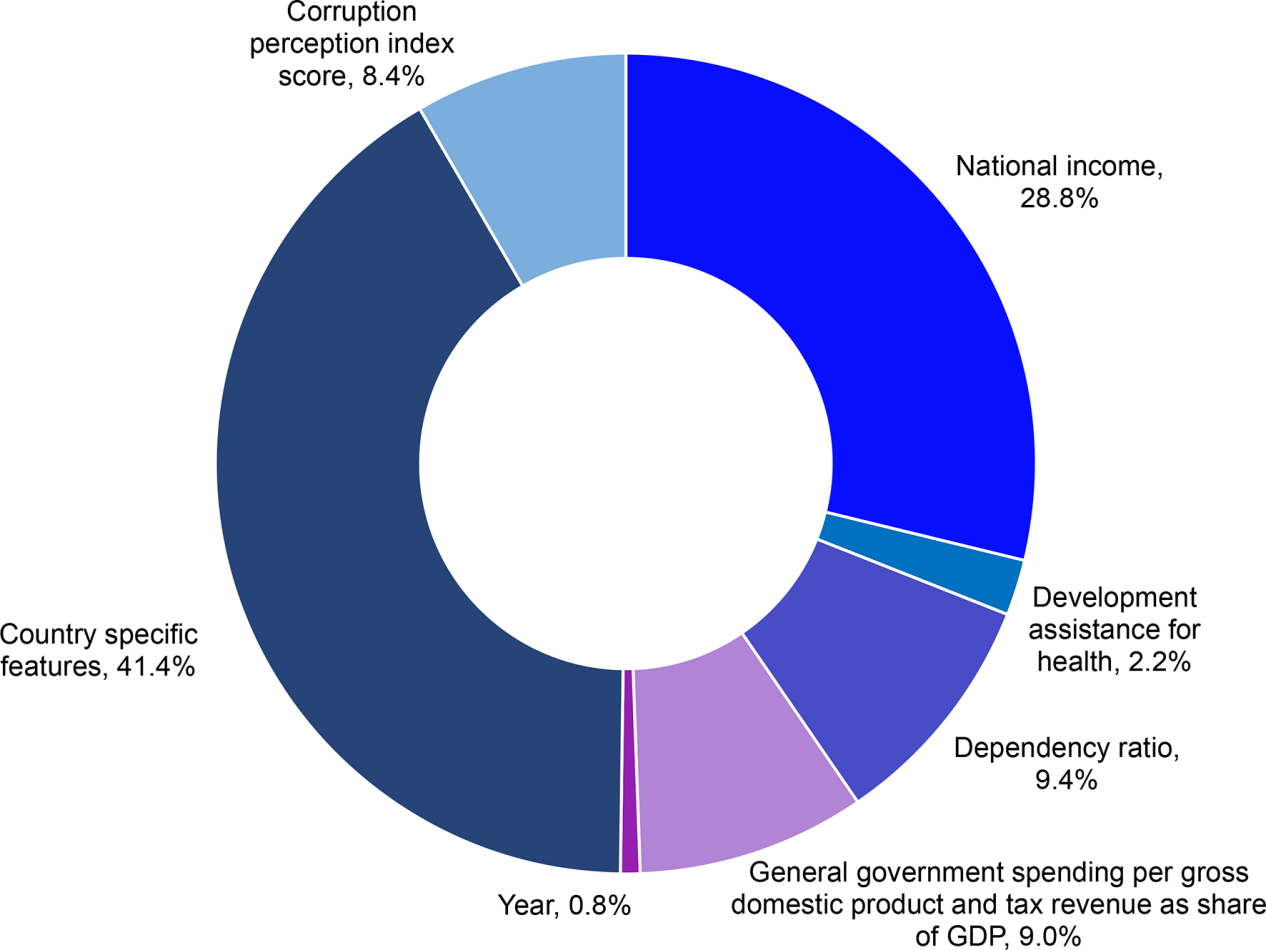
Decomposition of drivers of government health spending in sub-Saharan Africa: country-specific features, national income, development assistance for health, dependency ratio and general government spending per gross national product. GDP, gross domestic product.

Figure 2 describes the results of the decomposition analysis. Country-specific idiosyncrasies make up the highest share (41.41% [95% CI 39.32 to 43.09]) of the explained variation in government health spending. The perceptions of corruption in the public sector index explained 8.38% (95% CI 6.78 to 9.65). National income explained 28.80% (95% CI 27.12 to 29.75) of the variation in government health spending, while the dependency ratio explained 9.44% (95% CI 7.70 to 10.89) of the variation in government health spending. The two measures of government fiscal space—government spending as a share of national income and tax revenue as a share of national income—together explained 9.00% (95% CI 7.47 to 10.52) of the variation. The flow of development assistance for health and time trends explained the smallest amount of variation in health spending.

## Discussion

This study found that while there has been growth in overall government health spending since 1995, there are significant variations across countries in sub-Saharan Africa. Most interestingly, we found that while a country’s national income remained important as a determinant of government health spending, a country’s idiosyncrasies such as the perception of corruption in the public sector made up the highest share of the explained variation in government health spending in sub-Saharan Africa. These findings are critical because besides having some of the lowest ranking countries on the corruption index, also some of the lowest health spending globally is observed in countries in sub-Saharan Africa.

Among the countries in sub-Saharan Africa, government health spending grew at high rates during the years of the Millennium Development Goals (MDGs), in particular from 2000 through 2015. The focus of the MDGs on specific diseases such as HIV/AIDS and malaria and issues such as maternal and infant mortality that are observed at disproportionately high levels in African countries brought a great deal of attention and prioritisation to health on the continent. Subsequently, many African governments committed to meeting the goals set for these respective health areas. This commitment seems to have resulted in improved prioritisation and dedication of resources to health around that period. Nonetheless, despite the overall positive trends in growth rates, substantial variation persists. The fluctuation observed in growth in government health spending may be challenging for health system planners.

In this study, government health spending was measured relative to other factors including population, general government spending, size of the economy and total health spending. These different metrics provide relative comparisons and highlight the importance of context when comparing healthcare spending. Nonetheless, irrespective of the government health spending comparison, countries in Southern Africa seem to be among the largest spenders. It is important to note that countries in Southern Africa also have some of the highest burden of HIV/AIDS across the continent. The levels of government health spending suggest that their governments prioritised domestic public spending on health to address the epidemic in their countries.

Globally, $48.9 billion ($45.2–$54.2 billion) was spent on HIV/AIDS in 2015. The majority (61.0%, 95% uncertainty interval (UI) 55.1%–65.1%) of this total was spent by governments.^1^ In sub-Saharan Africa, the total spent on HIV/AIDS was $18.0 billion ($16.3–$20.5 billion), of which only 31.0% (24.6%–39.0%) came from governments; the majority ($3.9 billion, $2.5–$6.3 billion) of the total came from Southern Africa. East and Southern Africa are home to the largest number of people living with HIV globally.^35^ As such, in countries like South Africa and Botswana, where the AIDS epidemic was heightened, the cost of treatments such as antiretroviral therapy and the number of people infected compel large government expenditure envelopes.

This research also highlights that some of the countries whose governments spend the least on health are either countries in crisis or countries emerging from crisis. This crisis may be in the form of political unrest or natural disasters. For instance, in countries such as the Central African Republic, where ongoing strife compromises the normal running of government business, we observe very low government spending. Similarly, we also observe low government spending in Somalia, where a prolonged season of drought has further worsened living conditions.

A country’s total government health spending gives an idea of the volume of funds that the country has committed to health from the public purse, whereas government health spending per person puts that total volume in relation to the size of the population. This comparison reveals how this money translates to public spending on the health of each individual citizen. Examining government health spending as a share of general government spending allows us to see how governments in different countries prioritise health as a sector among the other competing demands on the public coffers. Viewing government health spending as a share of total health spending reveals to some extent what might be regarded as pooled spending in the various countries. Some studies have shown that universal health coverage is advanced in countries where pooled health spending makes up a larger share of total health spending.^36 37^ Measuring government health spending relative to the size of the country’s economy reveals the larger bucket from which resources are pulled. Mcintyre *et al* 38 have suggested that in order for countries to make good progress towards providing universal health coverage for all, countries must spend 5% of their GDP. In 2015, only Namibia had government health spending above that level. Suggestions for ways by which countries can improve their domestic health spending include improving efficiency in tax revenue collection, maximising tax collection from natural resource activities, and where applicable increasing tax rates.^39^

As determinants, both national income and general government spending as a share of the national income were positively associated with government health spending. The finding that the elasticity of government health spending with respect to gross national product is less than 1 suggests that government health spending is a necessity good. This finding is similar to that from other studies in both higher income and lower income countries on the determinants of government health expenditure.^4 5 19 20 32^ Improved economic conditions and strong economic growth are good things for the health sector. The larger the economic pie, the more of it there is to go around for all sectors. Improved resource mobilisation in terms of efficiency in tax revenue collection is an important strategy for increasing the fiscal space and thus increasing the economic pie in the country. Other studies have documented the importance of government revenue mobilisation efforts through various forms of taxes in driving growth in government health spending.^27–30^ We also find that in countries where the perception of corruption in the public sector is low, government health spending has increased. This finding is corroborated in another study where corruption is found to be associated with less government health spending in low-income and middle-income countries.^31^ Corrupt practices siphon limited public resources away from public goals. Whereas other studies have found results suggesting fungibility of domestic government health spending with the flow of external resources for health, this study did not find such a relationship. This could be because in this study we used a smaller sample set of countries and did not control for reverse causality, as was done in the other studies. In this study, we also did not find an association between the dependency ratio and domestic government spending on health. This finding is in line with what has been found in other similar studies.^20 21^ For countries in sub-Saharan Africa, as for other lower income countries, continued efforts to ensure and promote growth in the national economy remain an important strategy for making more resources available to promote better population health outcomes.

An important finding from this study is that individual country characteristics make up the highest percentage of the explained variation in government health spending across countries. Irrespective of a country’s income level, population structure or external resource flow, the country’s characteristics such as the perception of corruption in the public sector explain more of the level of government health spending observed in a country. This has important ramifications for ongoing endeavours intended to catalyse progress towards the SDGs. Interventions that find a way to promote country ownership of the goals may yield greater success in generating more spending on health from the government. It is important to note, however, that even with increased spending in some countries, their economic strength and the spending that additional commitment generates may still be insufficient to meet their health need or close the gaps between low-spending and high-spending countries.

This study has some limitations. While we are aware that income may have a bidirectional relationship with health, in that while economic prosperity may improve health, health spending may also promote economic prosperity, we have treated income in this study as having a unidirectional relationship with government health spending. This is acceptable in the context of this study because we did not aim to find causal determinants of government health spending. We were interested in understanding the associations of these factors with government health spending in order to determine their explanatory power for the variation in government health spending. We may have also omitted some variables. These include variables related to country health system characteristics such as health financing policies and disease burden. We believe such factors could also be potentially considered as outcomes of government health spending, and as such excluded them. If, however, these omitted variables are relevant in this context in a time-variant way, then our results may be biased.

## Conclusion

Attaining the health-related SDGs will require substantial resources. Whereas the MDGs coincided with a period of impressive growth in global health resources—in both government health spending and development assistance for health—we face a season of more tepid growth in development assistance for health resources in the SDG era. For countries in sub-Saharan Africa, as the attention of the global health community begins to focus on ways to generate and track domestic government health spending, the evidence here suggests that individual country sociopolitical characteristics such as perceptions of corruption in the public sector may be important in explaining whether countries are able to generate more resources for health.
