# Supplementary material for: Trends and drivers of government health spending in sub-Saharan Africa, 1995–2015
Source: BMJ Glob Health. 2019 Jan 13;4(1):e001159. doi: 10.1136/bmjgh-2018-001159 (PMC6350738; doi:10.1136/bmjgh-2018-001159)
Supplement: Supplementary data [file bmjgh-2018-001159supp001.pdf]

Annex

Transparency International Corruption Perception Index

Since 1995, Transparency International has produced the Corruption Perception Index (CPI), an index measure of the perceptions of corruption globally. The CPI is an aggregate of the perception of country experts and business people of corrupt practices in countries around the world. The most recently released – 2017 report- relied on surveys from 13 different sources and 12 different institutions that measure perceptions of corruption to generate estimates. It has a scale of 0 to 100, where 0 represents countries with the highest levels of corruption and 100 represents countries with the lowest levels of corruption. The measure captures perceptions of corruption in the previous two years. In 2012, the methodology was improved to make estimates historically comparable. In this analysis, to make earlier years of data comparable, reported values were scaled up by a factor of 10.

Source: https://www.transparency.org/news/feature/corruption_perceptions_index_2017#resources

Test 1: Hausman test – Fixed effects or Random effects model?

Test: Ho: difference in coefficients not systematic
chi2(7) = (b-B)'[(V_b-V_B)^(-1)](b-B)
= 31.60
Prob>chi2 = 0.0000
(V_b-V_B is not positive definite)

Conclusion: Use fixed effects.

Test 2: Inclusion of time fixed effects

F(20, 883) =	1.46
Prob > F =	0.0886

Conclusion: Time fixed effects inclusion not necessary but included in model to be conservative.

Test 3: Check for heteroskedasticity

Modified Wald test for groupwise heteroskedasticity
in fixed effect regression model
H0: sigma(i)^2 = sigma^2 for all i
chi2 (46) = 2471.08
Prob>chi2 = 0.0000

Conclusion: Modified Wald test for groupwise heteroskedasticity in fixed effect regression model indicates heteroskedasticity is present. Huber white sandwich estimators is implemented to correct using 'robust' option to get heteroscedasticity robust standard errors.

Test 4: Serial correlation

Wooldridge test for autocorrelation in panel data
H0: no first order autocorrelation
F(1, 45) = 216.840
Prob > F = 0.0000

.

Conclusion: Serial correlation test shows that we are unable to reject that we have first-order autocorrelation.

Unit root test results

IPS unit root tests					
Variable	L0	L0Trend	L1Trend	L2Trend	L3Trend
GDPpc	5.4469	1.8938	0.1187	0.5217	-0.4070
GHEpc	6.4184	5.9228	-0.7852	1.6528	n/a
GGE/GDP	-0.5900	-2.7011**	-0.6683	0.5719	-1.2153
DAHpc	-4.0447***	-7.5651***	-0.9132	0.0701	0.0200
Corruption perception Index	1.3153	1.7114	0.7093	0.7940	1.1529
Fisher-type ADF					
Variable					
GDPpc	95.4208	99.6046	135.4819***	98.2997	131.3327***
GHEpc	34.7977	34.6021	125.4375***	65.6173	88.4386
GGE/GDP	120.2086**	168.3624***	120.3653**	84.2195	150.9920***
DAHpc	182.4351***	314.6987***	151.1730***	117.3479**	164.1679***
Corruption perception Index	78.6978	69.5186	96.8922	138.2007***	118.8670**

Notes: *** significant at 1%; ** significant at 5%; *significant at 10%. For Im, Pesaran, and Shin unit root tests, t-tilde-bar values are presented. For Fisher-type ADF tests, inverse chi-squared statistics are presented. Natural logs of per capita values are used for macroeconomic variables. 'L' denotes the number of lags included and 'trend' refers to a test for trend-stationarity.
